# A study of neurologic symptoms on exposure to organophosphate pesticides in the children of agricultural workers

**DOI:** 10.4103/0019-5278.72242

**Published:** 2010-08

**Authors:** S. K. Rastogi, S. Tripathi, D. Ravishanker

**Affiliations:** Indian Institute of Toxicology Research, Lucknow, Uttar Pradesh, India; 1 Department of Biochemistry, Acharya Nagarajuna University, Andhra Pradesh, India

**Keywords:** Children, neurologic symptoms, OP pesticides

## Abstract

Pesticides are used extensively throughout the world in agriculture and in pest control as well as for community health purposes. Organophosphate (OP) pesticide self-poisoning is an important clinical problem in rural regions of the developing world that kills an estimated 200,000 people every year. Unintentional poisoning kills far fewer people but is an apparent problem in places where highly toxic OP pesticides are available. Neurologic dysfunction is the best documented health effect of pesticide exposure. High-level exposure has both acute and long-term neurologic signs and symptoms, and adverse effects have been reported in most type of pesticides, including organophosphate (OP), carbamate, organochlorine, and pyrethroid insecticides, herbicides, fungicides, and fumigants. Acute OP pesticide exposure can involve in wide range of both central and peripheral neurologic symptoms. Increased neurologic symptom prevalence may provide early evidence of neurologic dysfunctions, before clinically measurable signs are evident.

In this study, we analyzed the cross-sectional data on neurologic signs and symptoms from 225 rural children, both males (n = 132) and females (n = 93) who were occupationally and paraoccupationally exposed to methyl OPs (dichlorvos, fenthion, malathion, methyl parathion) and ethyl OPs (chlorpyrifos, diazinon, ethyl parathion) as they belonged to agricultural families handling, mixing, and spraying the OP pesticides. The children completed a specially designed questionnaire (Q16) on neurologic symptoms associated with pesticide exposure with their parental help. A suitable reference group consisting of rural children (n = 50) never involved in pesticide handling (neither outdoor nor indoor) belonging to similar socioeconomic strata included in the study to compare the prevalence of various neurologic symptoms between the two groups.

Among all the neurologic self-reported symptoms, headache, watering in eyes, and burning sensation in eye/face were the most important clinical manifestations attributed to OP pesticide exposure. These symptoms could probably be the consequence of chronic effects of most pesticides on the central nervous system. The muscarinic symptoms reported the maximum prevalence of salivation (18.22%), whereas lacrimation was observed in 17.33% cases, followed by diarrhea in 9.33% cases. The nicotinic clinical manifestations of acute OP poisoning revealed excessive sweating in 13.78% cases and tremors in 9.3% cases followed by mydriasis in 8.4% exposed children. The characteristic cholinergic symptoms, such as insomnia, headache, muscle cramps, weakness, and anorexia were also reported by both male and female exposed children. The high frequency of neurologic symptoms observed in the study may be due to parasympathetic hyperactivity due to the accumulated ACh resulting from AChE inhibition.

## INTRODUCTION

Organophosphate (OP) compounds are extensively used as pesticides and industrial chemicals. They are primarily neurotoxic and produce well-defined muscarinic, nicotinic, and cholinergic neurosymptoms involving both central and peripheral nervous systems.[1–3] Increases in both central and peripheral neurologic symptoms are also found in many studies on moderate exposure.[4] Increased symptom prevalence may provide early evidence of neurologic dysfunction, before clinically measureable signs are evident. High-level exposure has both acute and long-term neurologic effects, and adverse effects have been reported in most type of pesticides, including OP, carbamate, herbicides, and fungicides. OPs have been studied in great detail. Most previous studies of pesticides and neurologic symptoms have focused on OP pesticides. Farm workers,[5,6] greenhouse workers,[7] and pesticide factory workers[8] exposed to OPs reported more neurologic symptoms than unexposed workers. Similarly, farmers and farm workers who applied OPs had higher symptoms prevalence than did nonapplicators.[9–13]

## MATERIAL AND METHODS

A detailed cross-sectional health survey among the children from agricultural families in rural areas adjoining Lucknow, was carried out to assess the prevalence of chronic neurotoxicity, both OP-induced chronic neurotoxicity (OPICN) and OP-induced delayed neuropathy types, on the basis of self-reported neurologic symptoms and clinical examination. The questions on the symptoms were based on an established questionnaire Q16 that was used to evaluate the effects of occupational exposure to neurotoxicants by Lundberg *et al*.[14] The questionnaires are available on the AHS website.[15] The study involved with OP pesticide exposure and related morbidity on 225 rural children (both male and females) in the age group of 8–14 years from the adjoining areas, such as Malihabad, Mall, Rahimabad, Bakshi Ka Talab, Sitapur, and other neighboring villages, and the sample size taken from each study area, crops sprayed, and types of OPs sprayed in each respective area. A reference group of 50 children belonging to similar age group and socioeconomic strata selected from those rural families who had no family history of pesticides exposure served as the control group. The following parameters were studied during a health survey:

1. The general information regarding the age, weight, parental occupation, and frequency and duration of exposure to OP pesticides was recorded on a prestructured survey pro form (Q16).
2. Clinical examination with particular reference to neurologic system, symptom prevalence specific to chemical neurotoxicity, particularly irritability, insomnia, dizziness, anxiety, cholinergic toxicity, including SLUD sign (acronym for salivation, lacrimation, urination, and defecation), and muscarinic clinical manifestations on exposure to OP pesticides.

At enrollment, the participants completed a self-administered questionnaire that collected information on demographic characteristics, life style, medical history, and types and duration of pesticide use. All information on exposure and disease states were taken from these self-reports.

## RESULTS

Table 1 shows the area of study and the size of children population exposed to different types of OP pesticides directly or indirectly.

**Table 1:** Details of place and sample size from a health survey

Place of study	Sample size M/F = (n)	Pesticides exposure	Crops sprayed
Malihabad	24/17 = 41	Methyl parathion, malathion, chlorpyrifos methyl, ethion profenofos	Mango orchids
Mal	29/13 = 42	Dichlorvos, phorate, malathion, dimethoate, chlorpyrifos methyl, fenitrothion, diazinon	Mango orchids
Rahimabad	25/16 = 41	Chlorpyrifos methyl, dichlorvos, diazinon, methyl parathion, malathion, ethyl parathion	Mango orchids
Bakshi Ka Talab	31/28 = 59	Malathion, phosalone, ethion, chlorpyrifos methyl, phorate, dichlorvos	Mango orchids and vegetable, crops– potato, brinjal, tomato, rice, wheat
Sitapur	23/9 = 42	Chlorpyrifos, malathion, methyl parathion, dimethoate, phorate	Vegetable (potato, brinjal, tomato
Total	132/93 =225		

The range of age and period of exposure with reference to place of study is shown in Table 2.

**Table 2:** Place of study with reference to age and period of exposure

Place of study	Sample size (n) M/F = Total	Age range (years)	Range of exposure (years)
Malihabad	24/17 = 41	7–14	2–3
Mal	29/13 = 42	6–14	1–3
Rahimabad	25/16 = 41	9–14	1–2
Bakshi Ka Talab	31/28 = 59	8–13	1–4
Sitapur	23/19 = 42	7–14	2–4

The prevalence of OP pesticide–related symptoms observed in exposed rural children are illustrated in Table 3. The predominant symptoms associated with environmental exposure to pesticides were headache (8.44%), burning sensation in the eyes (10.22%), nausea and vomiting (7.11%), and dizziness (8.0%).

**Table 3:** Prevalence of pesticide-related symptoms

Symptoms	Control (n = 50) n (%)	Exposed population		
		Male = 132 n (%)	Female= 93 n (%)	Total= 225 n (%)
Headache	2 (4)	12 (9.09)	7 (7.53)	19 (8.44)
Burning sensation in eyes/face	3 (6)	18 (13.63)	5 (5.38)	23 (10.22)
Weakness	2 (4)	2 (1.52)	0 (0)	2 (0.88)
Fever	5 (10)	14 (10.61)	4 (4.30)	18 (8.00)
Watering eyes	1 (2)	16 (12.12)	5 (5.38)	21 (9.33)
Blurred vision	2 (4)	8 (6.06)	3 (3.23)	11 (4.89)
Skin irritation/itching	2 (4)	3 (2.27)	4 (4.30)	7 (3.11)
Dizziness	1 (2)	12 (9.09)	6 (6.45)	18 (8.00)
Nausea and vomiting	1 (2)	11 (8.33)	5 (5.38)	16 (7.11)
Breathlessness/chest pain	0 (0	5 (3.78)	2 (2.15	7 (3.11

The clinical manifestation of acute OP poisoning (muscarinic) reported by the pesticides exposed children are elaborated in Table 4. The maximum prevalence of excessive salivation was found in 18.22% cases, whereas lacrimation was observed in 17.33% exposed children. Miosis (4.9%), emesis (8.4%), and bronchoconstriction (5.7%) were the other cardinal symptoms observed in the study population.

**Table 4:** Clinical manifestation of acute organophosphate poisoning (muscarinic) among farm workers

Symptoms	Control (n = 50) n (%)	Exposed population		
		Male = 132 n (%)	Female = 93 n (%)	Total = 225 n (%)
Diarrhea	4 (8)	15 (11.36)	6 (6.45)	21 (9.33)
Urinary incontinence	3 (6)	2 (1.52)	1 (1.08)	3 (1.33)
Miosis	1 (2)	8 (6.06)	3 (3.23)	11 (4.9)
Bradycardia	0 (0)	2 (1.52)	0 (0)	2 (0.9)
Bronchoconstriction	0 (0)	7 (5.30)	6 (6.45)	13 (5.7)
Excessive salivation	1 (2)	31 (23.49)	10 (10.75)	41 (18.22)
Lacrimation	4 (8)	25 (18.93)	14 (15.05)	39 (17.33)
Emesis	5 (10)	11 (8.33)	8 (8.60)	19 (8.4)
Hypotension	0 (0)	3 (2.27)	1 (1.08)	4 (1.7)

The nicotinic clinical manifestations of acute OP poisoning are illustrated in Table 5. Excessive sweating was observed in 13.78% cases and mydriasis was observed in 8.4% cases. Some of the other notable symptoms found were tremors (9.3%), muscle weakness (5.7%), and tachycardia (3.1%) cases.

**Table 5:** Clinical manifestation of acute organophosphate poisoning (nicotinic) among farm workers

Symptoms	Exposed population		
	Male = 132 n (%)	Female = 93 n (%)	Total = 225 n (%)
Tremors	14 (10.61)	7 (7.53)	21 (9.3)
Muscle weakness	8 (6.06)	5 (5.38)	13 (5.7)
Hypertension	2 (1.52)	0 (0)	2 (0.8)
Tachycardia	4 (3.03)	3 (3.23)	7 (3.1)
Sweating	18 (13.63)	13 (13.98)	31 (13.78)
Mydriasis	13 (9.85)	6 (6.45)	19 (8.4)

No symptoms were observed in the controls

The long-term symptoms of OPICN in children exposed to OP pesticides (acute cholinergic toxicity) are briefed in Table 6. Some of the cholinergic symptoms, such as insomnia, headache, anorexia, and numbness in the lower limbs, were reported by both male and female children as a result of chronic exposure to OP pesticides.

**Table 6:** Long-term symptoms of OP-induced chronic neurotoxicity in children exposed to OP pesticides/symptoms of acute cholinergic toxicity

Symptoms	Male	Female	Over all
	(n = 132) n (%)	(n = 93) n (%)	(n = 225) n (%)
Tremors	13 (9.85)	8 (8.60)	21 (9.33)
Insomnia	3 (2.27)	2 (2.15)	5 (2.22)
Numbness in legs	5 (3.79)	4 (4.30)	9 (4.00)
Fatigue	8 (6.06)	5 (5.38)	13 (5.78)
Anorexia	11 (8.33)	7 (7.52)	18 (8.00)
Lethargy	9 (6.82)	6 (6.45)	15 (6.67)

## DISCUSSION

In this study, we found that increased neurologic symptom count was associated with chronic exposure to OP pesticides thereby confirming previous studies.[5–7] From the findings of this study, we can state that the principal morbidity in the children of agricultural workers consists of a higher incidence of neurologic disorders. Some of the most important clinical neurologic manifestations, such as headache, tremors, dizziness, and paresthesia, maybe attributable to and could be the consequence of the chronic effects of most of the OP pesticides on the peripheral and central nervous systems.[16] The pesticide-related neurologic symptoms, both muscarinic and nicotinic observed in this study could be due to the inhibition of RBCs, AChE, as well as plasma BuChE recorded in the study group and reported in earlier studies by the authors.[17,18] The acute cholinergic syndrome, such as miosis, excessive salivation, seizures, and others, could be due to overstimulation of postsynaptic acetylcholine accumulation resulting from AChE and BuChE inhibition by OP pesticides. Similar findings have also been reported by Saadeh *et al*[19] and Holstege and Baer.[20] Lotti[21] observed that the association between OP pesticide exposure and neurotoxic effects are well known. Farhat *et al*[22] also studied the association between pesticide exposure and neurologic health endpoints and concluded that environmental and occupational exposure to OP pesticides leads to neurodegerative functions in agricultural workers Studies on neurologic symptoms among Sri Lankan farmers showed that 24% of the neurologic symptoms resulted from occupational exposure to AChE-inhibiting OP insecticides.[23]

The long-term neurologic symptoms of OPICN resulting from cholinergic toxicity observed in the study population may be attributed to delayed toxicity by the OP pesticides as the breakdown of these chemicals in the body is slow and this results in the storage of pesticides in the body fat. Some of the OPs, such as diazinon and methyl parathion have significant lipid solubility, allowing fat storage with delayed toxicity due to late release.[24] Delayed toxicity may also occur atypically with other OPs, especially dichlorvos, fenthion, and demeton-methyl.[13]

The neurotoxic effects of OP pesticides could be related to conversion of thions (P=S) to oxons (P=O) in the body by the action of liver microsomes, which are more toxic than thions. The other possible mechanism that may lead to delayed neurotoxicity includes damage to the afferent fibers of peripheral and central nerves and associated with the inhibition of neuropathy target esterase. This delayed syndrome has been termed organophosphate induced delayed neuropathy, and manifested chiefly by weakness or paralysis and paresthesia of the extremities.

## CONCLUSIONS

We found that the prevalence of neurologic symptoms among the children of agricultural workers was associated with cumulative exposure (chronic) to OP pesticides used by this study population. These results suggest that self-reported neurologic symptoms involved both central and peripheral nervous systems, resulting from indoor and outdoor environmental exposure to OP pesticides.
